# Novel Pyropheophorbide Phosphatydic Acids Photosensitizer Combined EGFR siRNA Gene Therapy for Head and Neck Cancer Treatment

**DOI:** 10.3390/pharmaceutics13091435

**Published:** 2021-09-09

**Authors:** Chia-Hsien Yeh, Juan Chen, Gang Zheng, Leaf Huang, Yih-Chih Hsu

**Affiliations:** 1 Department of Bioscience Technology, Chung Yuan Christian University, Taoyuan 32023, Taiwan; super791031@hotmail.com; 2 Princess Margaret Cancer Center, University Health Network (UHN), Toronto, ON M5G 1L7, Canada; juanchen@uhnresearch.ca (J.C.); gang.zheng@uhnresearch.ca (G.Z.); 3 Department of Medical Biophysics, University of Toronto, Toronto, ON M5S 1A1, Canada; 4 Division of Pharmacoengineering and Molecular Pharmaceutics, Eshelman School of Pharmacy, University of North Carolina at Chapel Hill, Chapel Hill, NC 27599, USA; leafh@email.unc.edu; 5 Center of Cancer Theranostics and Commercialization of Technology, Chung Yuan Christian University, Taoyuan 32023, Taiwan; 6 Center for Nanotechnology, Chung Yuan Christian University, Taoyuan 32023, Taiwan

**Keywords:** targeted delivery, head and neck cancer, nanoparticles, photodynamic therapy

## Abstract

This study combined two novel nanomedicines, a novel LCP Pyro PA photodynamic therapy (PDT) and LCP EGFR siRNA gene therapy, to treat head and neck cancer. A novel photosensitizer, pyropheophorbide phosphatydic acids (Pyro PA), was first modified into Lipid-Calcium phosphate nanoparticles named LCP Pyro PA NPs, and targeted with aminoethylanisamide as a novel PDT photosensitizer. EGFR siRNA was encapsulated into LCP NPs to silence EGFR expression. Measured sizes of LCP EGFR siRNA NPs and LCP Pyro-PA NPs were 34.9 ± 3.0 and 20 nm respectively, and their zeta potentials were 51.8 ± 1.8 and 52.0 ± 7.6 mV respectively. In vitro studies showed that EGFR siRNA was effectively knocked down after photodynamic therapy (PDT) with significant inhibition of cancer growth. SCC4 or SAS xenografted nude mice were used to verify therapeutic efficacy. The LCP Control siRNA+PDT group of SCC4 and SAS showed significantly reduced tumor volume compared to the phosphate buffered saline (PBS) group. In the LCP-EGFR siRNA+LCP Pyro PA without light group and LCP EGFR siRNA + PBS with light group, SCC4 and SAS tumor volumes were reduced by ~140% and ~150%, respectively, compared to the PBS group. The LCP EGFR siRNA+PDT group of SCC4 and SAS tumor volumes were reduced by ~205% and ~220%, respectively, compared to the PBS group. Combined therapy showed significant tumor volume reduction compared to PBS, control siRNA, or PDT alone. QPCR results showed EGFR expression was significantly reduced after treatment with EGFR siRNA with PDT in SCC4 and SAS compared to control siRNA or PDT alone. Western blot results confirmed decreased EGFR protein expression in the combined therapy group. No toxic results were found in serum biomarkers. No inflammatory factors were found in heart, liver and kidney tissues. Results suggest that the novel LCP Pyro PA mediated PDT combined with LCP siEGFR NPs could be developed in clinical modalities for treating human head and neck cancer in the future.

## 1. Introduction

**Scheme 1:**
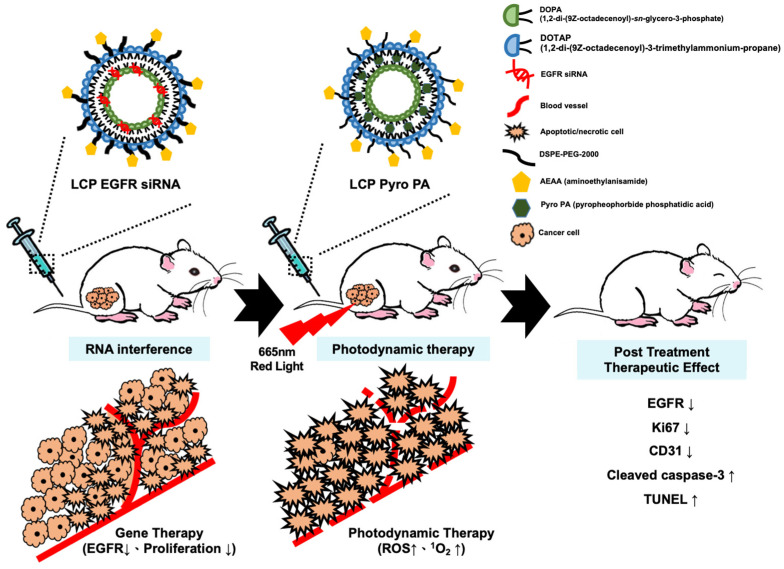
Schematic illustration of LCP EGFR siRNA and LCP Pyro PA to xenograft models. We used SCC4 and SAS xenografts of Balb/c nude mice to verify the efficacy of the combination therapy. Combined therapy showed greater tumor growth inhibition for both SCC4 and SAS.

Head and neck cancers develop mainly in the human oral cavity [1], larynx, hypopharynx and sinonasal areas. Ninety-five percent of head and neck cancer is caused by squamous cell carcinoma (SCC) [2]. Major risk factors include tobacco, betel nut chewing, alcohol drinking and HPV infections, which are the four major causes for carcinogenesis [2]. Clinical practices to treat human head and neck cancers include surgery, chemotherapy and radiotherapy. Another new modality is photodynamic therapy (PDT), a noninvasive alternative for oral cancer therapy with proven low cumulative side effects after repeated therapies, including little to no observed scarring in the oral cavity [3,4]. PDT uses a specific photosensitizer activated by a specific light wavelength that selectively kills cancerous cells [5]. Photodynamic reactions generate reactive oxygen species that can kill cancer cells. In this study, we are the first to develop a novel Lipid-Calcium phosphate–pyropheophorbide phosphatydic acids nanoparticles (LCP-Pyro PA NPs) technology. LCP Pyro PA NPs consist of LCP encapsulated with a novel photosensitizer, Pyro PA, with targeting ligand aminoethylanisamide (AEAA) on the outer-left layer of LCP NPs. Previous studies have found evidence of EGFR overexpression in human head and neck SCC, and oral cancers have been reported to demonstrate evidence of mRNA expression for EGFR [6,7]. Up-regulation of EGFR by squamous epithelial cells from 24 HNSCC patients with head and neck squamous cell carcinoma (HNSCC) suggests that therapies targeting these genes may be effective in the prevention of head and neck cancer [8]. We delivered small interfering RNA (siRNA) to target genes with specific siRNA to tumor cells without distinct adverse effects. The concept has been proven with transfected HIF1a siRNA [9] and VEGF siRNA [10] and induces in vitro cancer cell apoptosis and in vivo tumor destruction. Therefore, we delivered EGFR siRNA to head and neck tumor cells using lipid-based LCP NPs with AEAA targeting ligands formulated on the outer layer portion of the nanoparticles to target the over-expressed sigma receptors [11,12] on the HNSCC cell surface. The goal of this study was to investigate the therapeutic outcome of integrating a novel targeting photosensitizer, Pyro PA (LCP Pyro PA NPs mediated PDT), with LCP NPs with EGFR siRNA gene therapy and targeting AEAA ligands loaded to treat HNSCC in xenograft animal models (Scheme 1).

## 2. Materials and Methods

### 2.1. EGFR siRNA (siEGFR)

The EGFR siRNA (siEGFR) with sense strand 5′-AAG UGC UGG AUG AUA GAC GCA dTdT-3′ was designed using the provided Genome webserver (National Center for Biotechnology Information, Bethesda, MD, USA) [10]. The control siRNA (siControl) was the siGENOME non-targeting siRNA with sense strand 5′-AUGUAUUGGCCUGUAUUAGdTdT-3′ and was purchased from Thermo Scientific (Waltham, MA, USA). Lipofectamine^TM^ RNAiMAX was purchased from Invitrogen (Carlsbad, CA, USA). Dioleoylphosphatydic acid (DOPA), 1,2-dioleoyl-3-trimethylammonium-propane chloride salt (DOTAP), cholesterol and 1,2-distearoryl-*sn*-glycero-3-phosphoethanolamine-*N*-[methoxy(polyethyleneglycol-2000)] ammonium salt (DSPE-PEG2000) were all purchased from Avanti Polar Lipids (Alabaster, AL, USA). DSPE-PEG-AEAA was chemically synthesized in the Hsu lab [12]. Other chemicals used in the study were purchased from Sigma-Aldrich (St. Louis, MO, USA).

### 2.2. Novel Synthesis Method of Pyro PA Photosensitizer

Pyropheophorbide-phosphatydic acid (Pyro PA) was synthesized and provided by Professor Zheng (Toronto, ON, Canada). Forty mg of pyropheophrobide-phospholipid (Pyro-lipid) was suspended in 3 mL of sodium acetate buffer (40 mM sodium acetate, 80 mM CaCl_2_, pH 5.0) and sonicated for 5 min for efficient dispersion, after which 3 mL of diethyl ether and 1000 U of phospholipase D obtained from *S. chromofuscus* were added. The mixture was light sensitive and therefore was protected from light and mixed aggressively at 33 °C. The reaction was completed in 5 h to yield Pyro PA, evidenced by HPLC-MS analysis. The HPLC profiles of the Pyro-lipid and Pyro PA and their corresponding UV spectra and mass spectra were further examined and verified to ensure the success of the synthesis.

### 2.3. Novel Formulation of LCP Pyro PA NP

First, phosphatydic acid was conjugated into Pyro PA for encapsulating into the lipid calcium phosphate nanoparticles. Then, 100 µL of 0.5 M CaCl_2_ was dispersed in an 8 mL oil phase (cyclohexane/Igepal CO-250 [71/29, *v*/*v*]) to form a homogenous water-in-oil well-dispersed microemulsion. The phosphate micro-emulsion was prepared by dispersing 100 µL of 100 mM Na_2_HPO_4_ in a separate 8-mL oil phase (cyclohexane/Igepal CO-250 [71/29, *v*/*v*]). Concentrations of 17.3 mM DOPA and 17.3 mM Pyro PA were dissolved in chloroform and added to the phosphate micro-emulsion solution. Then, the two micro-emulsions were mixed for 30 min, and ethanol (16 mL) was added to the combined solution. The well-mixed solution was then centrifuged to remove unnecessary ingredients. After three repetitions of the ethanol wash, the CaP core pellets were collected as white precipitates. The final LCP NPs were prepared in a separate step: 50 µL of LCP CaP cores were mixed with 20 µL of 20 mM DOTAP, 20 mM cholesterol, 20 mM DSPE-PEG-AEAA, and 20 mM DSPE-PEG-2000. Finally, residual lipids were evaporated. LCP NPs were formed when rehydrated in 50 µL of 20% glucose solution for tail vein injection into mice. Aminoethylanisamide (AEAA) was modified on the outer leaflet of LCP NPs to target the sigma receptors that are overexpressed on HNSCC cells [13].

### 2.4. Formulation of LCP Nanoparticles 

LCP NPs were formulated as described with slight modifications [13,14,15,16]. Sigma receptors are highly expressed on the surface of SCC4 or SAS cell lines, and AEAA is the target moiety against sigma receptors on the surface of LCP NPs [12]. In an oil-phase solution (20 mL) comprising cyclohexane and Igepal CO-250 at 71/29 (*v*/*v*) ratio, a homogenous water-in-oil microemulsion solution was formed. Subsequently, 300 µL of 2.5 M CaCl_2_ with 60 µg of siRNA was dispersed into the microemulsion system. A phosphate microemulsion was formulated by adding 300 µL of 12.5 mM Na_2_HPO_4_ in a 20 mL oil phase bottle. One hundred microliters of 20 mM DOPA dissolved in chloroform solvent was transferred to the phosphate solution. After mixing the two microemulsions homogenously for 20 min, ethanol (40 mL) was added to the final solution and then centrifuged to spin down the LCP NPs. It was crucial to conduct the ethanol wash procedure 3–4 times, after which the CaP core pellets were collected. The final LCP NPs were prepared in a separate step; 50 µL of LCP CaP cores was mixed with 20 µL of 20 mM DOTAP, 20 mM cholesterol, 20 mM DSPE-PEG-AEAA, and 20 mM DSPE-PEG-2000. Finally, residual lipids were evaporated. LCP NPs were formed when rehydrated in 50 µL of 20% glucose solution for tail vein injection into mice.

### 2.5. Characterization of LCP Nanoparticles

Transmission electron microscope images of LCP were obtained using the Bio-TEM Hitachi HT7700 (Hitachi, Ibaraki, Japan). LCP NP size and zeta potential were determined using the Malvern Zetasizer Nano series (Westborough, MA, USA).

### 2.6. Human SCC Cell Cultures

The SCC4 cell line was acquired from the Bioresource Collection and Research Center (BCRC, Hsinchu, Taiwan) and the SAS cell line was a gift from Prof. Chun-Ji Liu, Yang Ming University. Both SCC4 and SAS cells were cultured using DMEM/F-12 medium (Invitrogen, Grand Island, NY, USA), supplemented with 10% fetal bovine serum (FBS) (Invitrogen, Grand Island, NY, USA) and incubated at 37 °C with 5% CO_2_. SCC4 and SAS cells were removed from culture plates using 0.05% trypsin-EDTA (Invitrogen, Carlsbad, CA, USA) when cancer cells reached 80% confluence on plate.

### 2.7. In Vitro siRNA Transfection Study

In a six-well plate, 3 × 10^5^ cells per well of SCC4 or SAS cells were cultured on each well and incubated for 24 h. Transfection was conducted with 25 nM siRNA concentration in Opti-MEM Reduced medium (Invitrogen, Grand Island, NY, USA) using Lipofectamine^TM^ RNAiMAX (Invitrogen, Carlsbad, CA, USA) based on manual. SCC4 or SAS cells were then placed into an incubator at 37 °C for 4 h in the Opti-MEM Reduced medium. The medium was then changed to a growth medium containing 10% FBS for 48 h, after which cells were lysated.

### 2.8. SCC4 and SAS Xenograft Model Establishment

Eight week old male BALB/cAnN.Cg-Foxn1 nude mice were purchased from the National Laboratory Animal Center, Taipei, Taiwan. To establish SCC4 or SAS xenograft mice models, 5 × 10^5^ cells of SCC4 or SAS in DMEM/F12 media were combined gently with matrigel (Corning, Bedford, MA, USA) and infused subcutaneously at the right side of the lower flank of each mouse. SCC4 and SAS xenograft mice were randomly divided into five groups with five mice in each group: (1) PBS; (2) LCP siControl NPs+PDT; (3) LCP Pyro PA NPs without light+LCP siEGFR; (4) LCP siEGFR NPs+PBS+light; and (5) LCP siEGFR NPs+PDT. The five treatments were given intravenously via tail vein infusion. The dosage of LCP siControl NPs and LCP siEGFR NPs was 0.36 mg/kg. The dosage of LCP Pyro PA NPs was 0.78 mg/kg, prepared by solubilizing 0.3 mg Pyro PA in 200 µL PBS for 25 g of weight and given via tail vein. After 55 min, red light at 663 ± 9 nm wavelength with 320 mW/cm^2^ power density delivered 100 J/cm^2^ energy dosage for 11 min. Tumor size reached 200 ± 5% mm^3^ (190~210 mm^3^) and was sufficient for treatment. The formula used to calculate tumor volume was V = (L × W × H)/2, where V stands for tumor volume, L stands for length, W stands for width perpendicular to length, and H stands for height of the tumor. Tumor size was determined daily using calipers and all mice were sacrificed on the 13th day for further analysis. Excised tumors and organs were divided and fixed in formalin for H&E and IHC stainings. This animal study was approved on August 6, 2015 (case number #103030) and carried out under strict guidelines based on the recommendations of the Guide for the Care and Use produced by the Institutional Animal Care and Use Committee of Chung Yuan Christian University, Chungli, Taoyuan, Taiwan.

### 2.9. Western Blot Experiments

Cells were harvested or tumor specimens removed and homogenized using a lysis buffer (PRO-PREPTM^TM^ protein extraction solution, Intron Biotechnology Inc., Seoul, Korea). These samples were assayed. Identical amounts of protein determined using BCA protein assays (Pierce, Thermo Fisher Scientific Inc., Rockford, IL, USA) were assayed for these specimens. Lysated cells or tissues were first heated to denatured soluble proteins in the sample buffer at 100 °C for 5 min. Then, these prepared specimens were loaded in individual wells of 5%/12% Bis-Tris acrylamide gels (stacking/separating gel) with protein markers. SDS-PAGE electrophoresis was conducted at a constant voltage (150 V) at 25 °C. Proteins on the SDS-PAGE gels were transferred to a PVDF membrane (Millipore Corporation, Billerica, MA, USA) electrophoretically at a constant voltage for 2 h at 4 °C. After the protein transfer step, the PVDF membranes were blocked with a blocking buffer (BlockPROTM^TM^, Visual Protein Biotechnology Corporation, Taipei, Taiwan), then each fraction of the membranes was separated and incubated with rabbit primary antibodies against EGFR (GTX121919; GeneTex, Taipei, Taiwan) (1:1000 dilution) and sigma receptor (SIGMAR1 GTX115389; GeneTex, Taipei, Taiwan) (1:1000 dilution), followed by peroxidase-conjugated goat anti-rabbit IgG (GTX213110 GeneTex, Taipei, Taiwan) (1:10,000 dilution), and then developed in ELC (enhanced chemiluminescence) substrate (PerkinElmer, Inc., Boston, MA, USA). Glyceraldehyde 3-phosphate dehydrogenase (GAPDH GTX100118; GeneTex, Taipei, Taiwan) (1:1000 dilution) was used as the internal control.

### 2.10. Quantitation of EGFR Gene Knock-Down Using Real Time-PCR

First, RNA was extracted from the tumor tissues. Tumor specimens were divided into small pieces and several pieces of 0.05 g were randomly chosen from each experimental group. Tumor specimens were homogenized for extraction of total tissue RNA using RNAzol^®^ RT solution (Molecular Research Center Inc., Cincinnati, OH, USA). The obtained cDNAs were synthesized using RevertAid cDNA synthesis kits (Fermentas, Thermo Scientific Inc., Waltham, MA, USA). FastStart Universal Master Probe (Roche Applied Science, Mannheim, Germany) was used for quantitative real-time PCR. PCR were operated using a regular cycling schedule: 95 °C for 10 min, 40 cycles of 95 °C for 15 s and 60 °C for 1 min on a real-time PCR system (AB7300, Applied Biosystems, Foster City, CA, USA). The forward primer sequences for human EGFR were 5′-TTCCTCCCAGTGCCTGAA-3′and the reverse primer sequences for human EGFR were 5′-GGGTTCAGAGGCTGATTGTG-3′. The forward primer sequences for human GAPDH were 5′-AGCCACATCGCTCAGACAC-3′ and the reverse primer sequences for human EGFR were 5′-AGCCACATCGCTCAGACAC-3′. The forward and reverse primer pairs were produced by Roche (Roche Applied Science, Mannheim, Germany). Calculated results were based on the relative times of threshold schedules in which the calculated EGFR concentration data were normalized against GAPDH concentration as internal control.

### 2.11. H&E Staining and Immunohistochemistry (IHC)

Harvested organs and tumor tissues were embedded in paraffin and divided into several connective sections followed by standard H&E staining protocol. Tissue sections of paraffin-embedded SCC4 and SAS tumors were deparaffinized and rehydrated to retrieve epitopes on the antigens for IHC. Tissue slides were treated with either monoclonal or polyclonal antibodies, such as rabbit polyclonal anti-CD31 (1:100, ab28364, Abcam, Cambridge, MA, USA), rabbit monoclonal anti-Ki-67 (1:200, ab16667, Abcam, Cambridge, MA, USA), and rabbit polyclonal anti-EGFR (1:100, GTX121919; GeneTex, Taipei, Taiwan). Sections of tumor tissues were then incubated with HRP-conjugated anti-rabbit antibody (1:200, Santa Cruz Biotechnology, Santa Cruz, CA, USA) for 30 min. Slide observation was conducted using a DAB detection kit (Pierce, Rockland, IL, USA). All specimens were examined using an Olympus light microscope (BX53F model, Olympus, Tokyo, Japan). Intensities of IHC stains were further quantified for comparison at 40× magnification (10 images per group) using ImageJ software on 21 July 2016 (version 1.8, National Institutes of Health, Bethesda, MD, USA, http://imagej.nih.gov/ij/).

### 2.12. TUNEL Assays

SCC4 and SAS tumor paraffin-embedded tissue sections were deparaffinized, rehydrated and pretreated for protease. TUNEL assays was executed using in situ Cell Death Detection Kits, POD (Roche, Mannheim, Germany) following the manufacturer’s guidelines. Specimens were examined using an Olympus BX53F light microscope (Olympus, Tokyo, Japan). Positive TUNEL cell images were recorded and quantified at 40× magnification using ImageJ software (National Institutes of Health, http://imagej.nih.gov/ij/).

### 2.13. In Vivo Toxicity Assays

In vivo toxicity assays followed the same protocol as treatment for tail vein infusion of four treated groups including LCP NPs of PBS, siControl, siEGFR, and Pyro PA. Three mice were used in each group. The 7- to 8-week-old C57BL/6JNarl mice were tail vein injected with PBS, LCP siControl NPs, and LCP siEGFR NPs on days 0, 1 and 2, with injections repeated on days 7, 8 and 9, and a 4-day interval between injections (of three experiment groups). The LCP Pyro PA NPs group was also tail vein injected into mice on days 3 and 10. Mice were anesthetized and cardiac punctured to collect blood for toxicity assays and sacrificed on the 14th day. To avoid hemolysis of mouse blood, blood was carefully transferred to a 1.5 mL vial in a slow mode, and blood was set to clot at 25 °C for 20 min prior to centrifugation to separate serum from the supernatant portion (Hermle Z233 MK-2, Hermle, Wehingen, Germany). The collected serum was analyzed for concentrations of liver biomarkers (AST and ALT), kidney biomarkers (CREA and BUN), calcium, and phosphorus. Concentrations of toll-like receptor 3 (TLR3) were also examined using TLR3 ELISA kits (MyBioSource, Inc., San Diego, CA, USA) to ensure no immune activation. The above assays used mouse IL-6 ELISA kits (RayBiotech, Norcross, GA, USA), mouse IL-12 p40/70 ELISA kits (RayBiotech, Norcross, GA, USA), mouse IFN gamma ELISA kits (RayBiotech, Norcross, GA, USA) and TLR3 ELISA kits (MyBioSource Inc., San Diego, CA, USA), respectively.

### 2.14. Statistical Analysis

All data were presented as mean ± standard deviation. Statistical significance was analyzed using one-way ANOVAs with Tukey’s test using SigmaPlot^®^ (Version 12.5, Systat Software, Inc., San Jose, CA, USA).

## 3. Results

### 3.1. Results of Novel Synthesis Method of Pyropheophrobide-Phosphatydic Acid (Pyro Pa) from Pyropheophrobide-Phospholipid (Pyro-Lipid)

**Figure 1:**
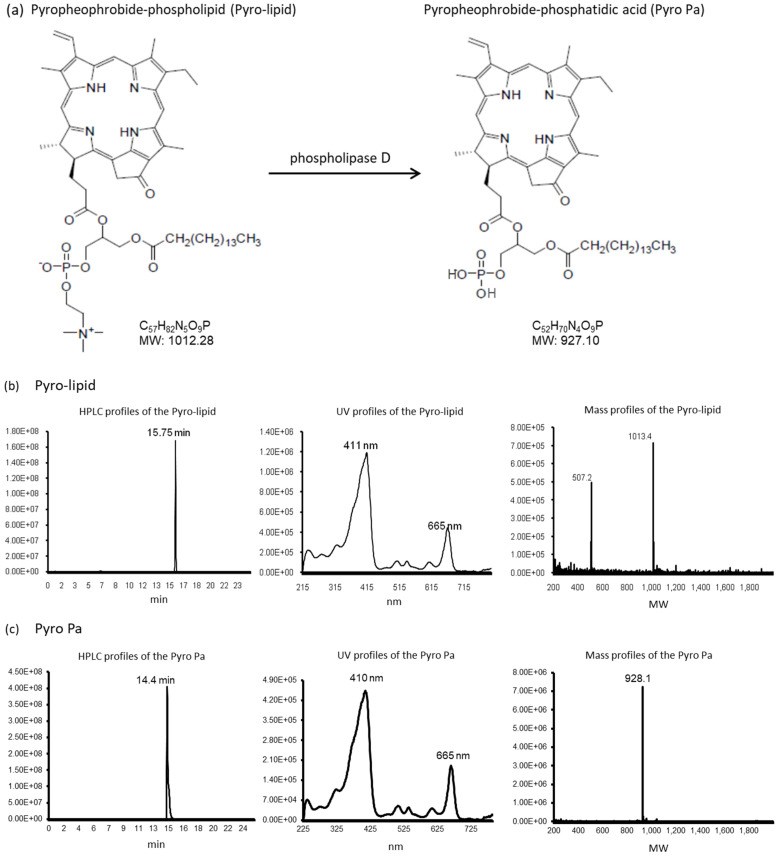
Novel synthesis method of Pyro PA. (**a**) 40 mg of pyropheophrobide-phospholipid (Pyro-lipid) was suspended in 3 mL of sodium acetate buffer (40 mM sodium acetate, 80 mM CaCl_2_, pH 5.0) and sonicated for 5 min for efficient dispersion, after which 3 mL of diethyl ether and 1000 unit of phospholipase D obtained from *S. chromofuscus* were added. The mixture was shaded against light and mixed extensively at 33 °C. (**b**,**c**) The reaction was completed in 5 h to gain pyropheophrobide-phosphatydic acid (Pyro PA) evidenced by HPLC-MS analysis. The HPLC profiles of the Pyro-lipid and Pyro PA and their corresponding UV spectra and mass spectra were examined and verified to ensure success.

The innovative synthesis of pyropheophrobide-phosphatydic acid (Pyro Pa) from pyropheophrobide-phospholipid (Pyro-lipid) used phospholipase D (from *S. chromofuscus*) as described in the Section 2 (Figure 1a). The reaction was completed to gain Pyro PA, as evidenced by HPLC-MS analysis (Figure 1b,c). The figures show HPLC profiles of the Pyro-lipid (Figure 1b) and Pyro PA (Figure 1c) and their corresponding UV spectra and mass spectra. The compound absorption spectrum had no significant changes during the reaction. The identified mass signals for Pyro-lipid were: *m*/*z* calculated for C_57_H_82_N_5_O_9_P [M]^+^ 1012.3, found [M]^+^ 1013.2; [M]^2+^ 507.2; for Pyro PA: *m*/*z* calculated for C_52_H_70_N_4_O_9_P [M]^+^ 927.1, found 928.1.

### 3.2. Characterization of Lipid-Calcium-Phosphate Pyropheophrobide Phosphatydic Acid Nanoparticles (LCP Pyro Pa NPs) or Lipid-Calcium-Phosphate EGFR siRNA Nanoparticles (LCP EGFR siRNA NPs)

#### 3.2.1. Preparation of Lipid-Calcium-Phosphate Pyropheophrobide Phosphatydic Acid Nanoparticles (LCP-Pyro Pa NPs)

**Figure 2:**
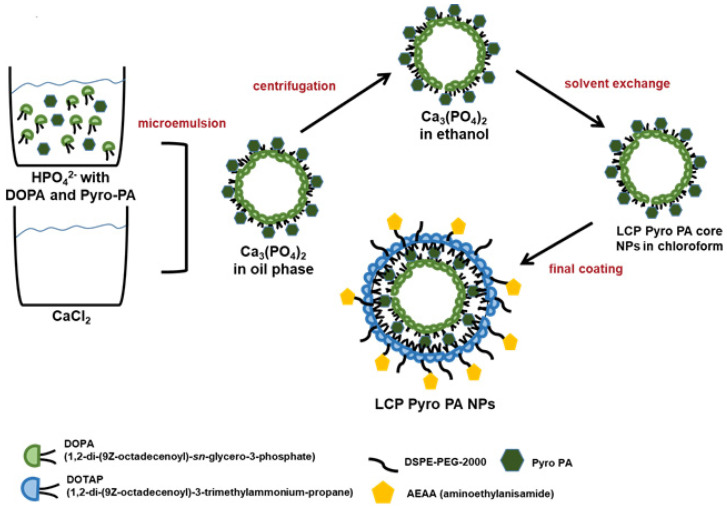
Novel synthesis method of LCP Pyro-Pa nanoparticles. LCP Pyro-Pa NP was composed of a biodegradable calcium phosphate core stabilized using DOPA and DOTAP. The outer layer of lipid was modified with polyethylene glycol (PEG) chains and aminoethylanisamide.

LCP-Pyro PA NPs were formulated with two different types of phospholipids to form double-layer liposomal nanoparticles that were composed of pH-sensitive calcium phosphate cores stabilized by DOPA and coated with DOTAP. The outer part of the lipid layer was modified with polyethylene glycol (PEG) chains and aminoethylanisamide (AEAA). AEAA is the targeting ligand for sigma receptors on cancer cell surfaces (Figure 2a). TEM photomicrographs indicated that nanoparticles were 15 to 20 nm in size. The zeta potential determined for LCP Pyro Pa NPs was 52.0 ± 7.6 mV with AEAA and 3.5 ± 0.6 mV without AEAA (Figure 2).

#### 3.2.2. Preparation of Lipid-Calcium Phosphate EGFR siRNA Nanoparticles (LCP EGFR siRNA NPs)

**Figure 3:**
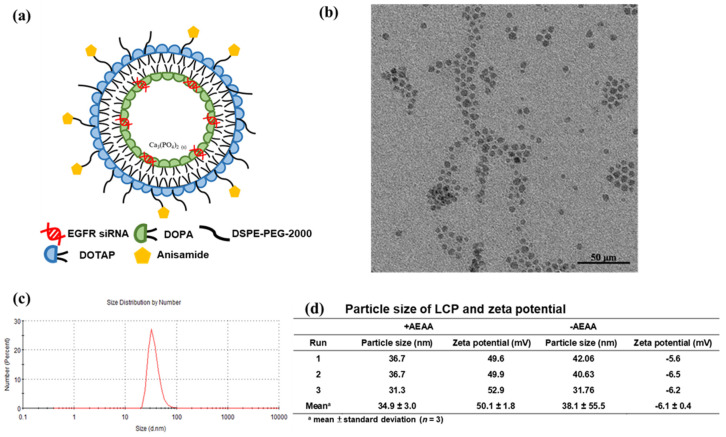
Characteristic Properties of LCP siEGFR NPs. (**a**) Demonstration of LCP siEGFR NPs. LCP Pyro PA NPs were formulated with two different types of phospholipids to form double-layer liposomal nanoparticles that were composed of pH sensitive calcium phosphate cores stabilized by DOPA and coated with DOTAP. The outer part of the lipid layer was modified with polyethylene glycol (PEG) chains and aminoethylanisamide (AEAA). AEAA is the targeting ligands for the sigma receptors on cancer cell surfaces (**b**–**d**). Data presented as mean ± SD (in triplicate).

Refer to the similar descriptions shown above for LCP-Pyro PA NPs (Figure 3a). As TEM was conducted in dehydrated conditions, it showed nanoparticle sizes ranging from 25 to 30 nm. The LCP NP sizes detected by dynamic light scattering technology were larger than the TEM-measured NP sizes, from 31 to 37 nm, because of the hydrodynamic diameter. The zeta potential for LCP siEGFR NPs was 50.1 ± 1.8 mV with AEAA and −6.1 ± 0.4 mV without AEAA. Data were presented as mean ± SD (in triplicate) (Figure 3b–d).

### 3.3. In Vitro Sigma Receptor and EGFR Protein Expression

**Figure 4:**
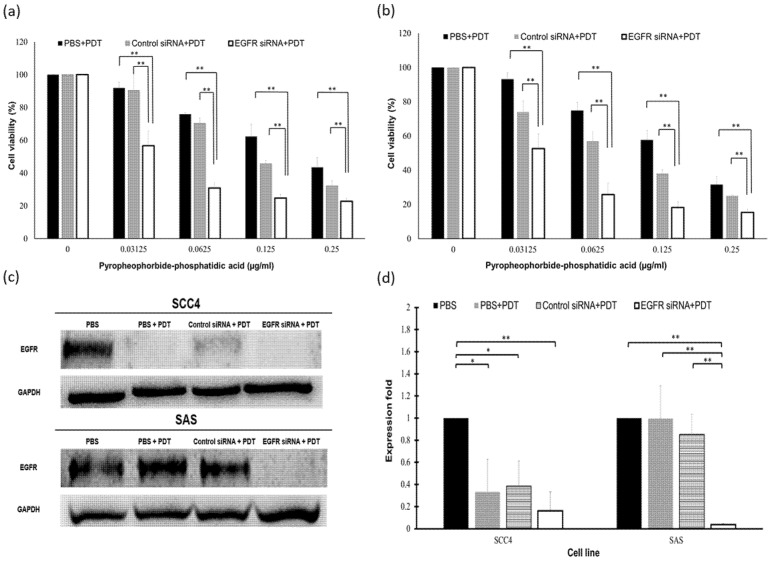
In vitro study of combined therapy. Combined therapy of EGFR siRNA or Control siRNA and photodynamic therapy (PDT) with different concentrations of Photosan photosensitizers in SCC4 (**a**) or SAS (**b**) cell lines. (**c**,**d**) Western blots of EGFR protein level in siRNA treated SCC4 and SAS cells. Columns, mean (*n* = 3); * stands for *p* < 0.05 compared to PBS group. ** stands for *p* < 0.01 compared to PBS group.

Our past studies have reported that sigma receptors are expressed on the surface of HNSCC in vitro [9,10]. We examined the expression of sigma receptors and EGFR proteins at one day after photodynamic therapy. Either SCC4 or SAS cells were then treated with 0~0.25 μg /mL Pyro Pa NPs μg/mL irradiated with 663 ± 9 nm at 10 J/cm^2^ described as the PDT group in the in vitro study. Subsequently, they were incubated in the medium for 24 h. Cell viability was decreased due to the EGFR siRNA knockdown effect and follow-up PDT treatment in SCC4 (Figure 4a) and SAS (Figure 4b) cell lines. Either SCC4 or SAS cells were then transfected with 12.5 nM self-designed EGFR siRNA. After two days’ incubation, EGFR protein amount was examined using Western blots. An EGFR silencing effect was observed for SCC4 and SAS cells treated with EGFR siRNA+PDT as compared to the groups treated with phosphate buffered saline (PBS)+PDT or Control siRNA+PDT. This indicates that the novel EGFR siRNA sequence could knockdown EGFR expression (Figure 4c,d).

### 3.4. In Vivo Treatment Efficacy of Combination Therapy 

Two HNSCC xenograft models, SCC4 and SAS models of 204~209 mm^3^ tumor volume, were then randomly distributed to five study subgroups: (1) PBS; (2) PDT (LCP siControl+PDT); +LCP Control siRNA, (3) LCP-Pyro PA+LCP EGFR siRNA, (4) PBS+light+LCP EGFR siRNA and (5) PDT (LCP-Pyro PA+light)+LCP EGFR siRNA. The complete therapeutic protocol was conducted for 13 days. All mice received two treatments on days 0, 1, or 2 and 7, 8, or 9. Either photosensitizer or LCP NPs containing siControl or siEGFR were injected into mice via the tail vein. On days 3 and 10, the mice received either PBS or LCP Pyro PA NPs (2 mg/kg) and were given 663 ± 9 nm red light to reach 100 joules after a 55 min metabolic period. For further analysis, all mice were humanely anaesthetized to death on the 13th day to collect major organs and tumors.

**Figure 5:**
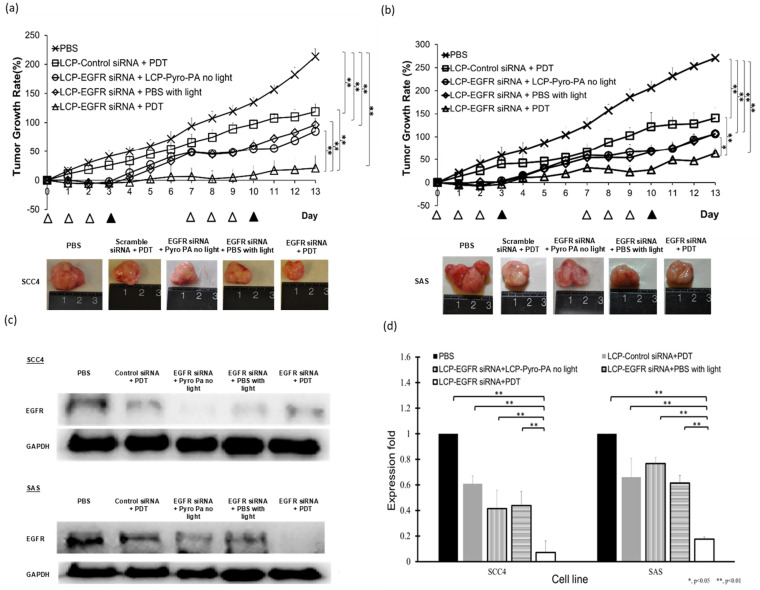
In vivo study of combined therapy. Tumor curves of SCC4 (**a**) and SAS (**b**) xenograft models. The experiments were conducted approximately 3–5 weeks post cell inoculation once the tumor volume reached approximately 200 mm^3^. △ indicated IV infusion of LCP with either Control siRNA or EGFR siRNA or PBS; ▲ indicated IV infusion of LCP Pyro PA with or without light. Tumor images of collected SCC4 (**a**) and SAS (**b**) xenograft tumors on day 13. (**c**) Quantified relative EGFR phosphorylated-EGFR (tyr1173) phosphorylated-EGFR (tyr1092) phosphorylated-EGFR (tyr1045), Her2, AKT, phosphorylated-AKT, cleaved caspase-3 and GAPDH protein expression band intensity in SCC4 and SAS cell line. (**d**) QPCR analysis of EGFR protein expression after treatment for SCC4 and SAS xenograft model. Quantified relative EGFR protein expression normalized against GAPDH. GAPDH protein was applied as the internal normalized protein marker with their respective relative EGFR protein expression. Columns, mean (*n* = 3); *, *p* < 0.05; **, *p* < 0.01.

The tumors of the PBS group increased in size by up to 213% and 270% after 13 days in the SCC4 and SAS xenograft models, respectively (Figure 5a,b). On day 13, the LCP siControl+PDT group showed decreased tumor size after the PDT treatments on days 3 and 10 with ~100% and ~145% inhibition of tumor volume in the SCC4 and SAS xenograft models, respectively. Both SCC4 and SAS models demonstrated significant differences (*p* < 0.01) when compared with PBS. On day 13, LCP siEGFR+PS and LCP siEGFR+PBS+light groups showed ~130% and ~175% inhibition of tumor volume in the SCC4 and SAS xenograft models, respectively. Both SCC4 and SAS groups showed tumor volume inhibition resulting from LCP siEGFR in the absence of a complete PDT treatment. The combined group, LCP Pyro PA NPs–mediated PDT, showed significant tumor volume inhibition after LCP siEGFR silencing as compared to the other four groups (*p* < 0.01); combined therapy demonstrated significant tumor volume decrease of ~200% and ~220% in the SCC4 and SAS models, respectively, when compared with untreated PBS groups, with significant differences for both models (*p* < 0.01).

Quantitative RT-PCR and Western blot tests were conducted to examine SCC4 and SAS tumor tissues obtained on the 13th day. Protein expression of EGFR for LCP siEGFR NPs groups decreased significantly (*p* < 0.01). All data were analyzed and normalized with GAPDH densities when compared with PBS and LCP siControl+PDT groups for SCC4 and SAS xenograft models (Figure 5c). EGFR expression was consistent with assayed siEGFR mRNA expression for SCC4 or SAS xenograft animal models (Figure 5d) and significant differences (*p* < 0.01) were found in LCP siEGFR NPs compared with PBS and the three other treated groups. 

### 3.5. Combination Therapy Does Inhibit HNSCC Tumor Growth Efficiently 

**Figure 6:**
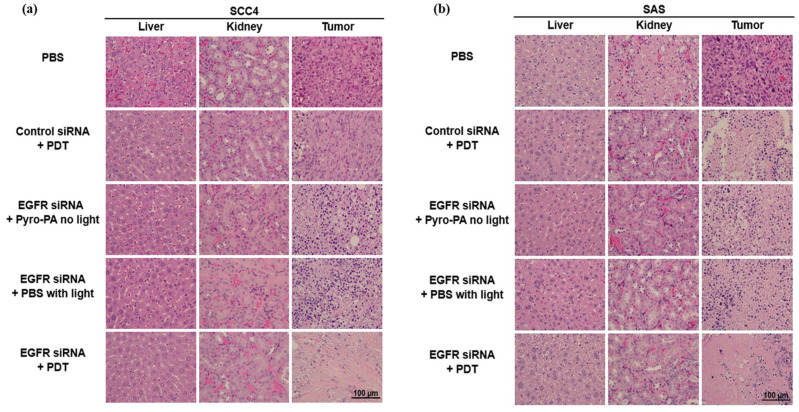
Hematoxylin and eosin staining of SCC4 and SAS Tumors. Hematoxylin and eosin histopathological stains of liver and kidney organs and SCC4 (**a**) and SAS tumors (**b**) of xenograft mice. Original magnification 40×. Scale bar stands for 100 μm.

Hematoxylin and eosin (H&E) staining assays for SCC4 or SAS tumors tissues obtained from xenografted mice after treatments were conducted to observe tumor, liver and kidney damage. No cell damage was found in livers or kidneys (Figure 6a,b).

**Figure 7:**
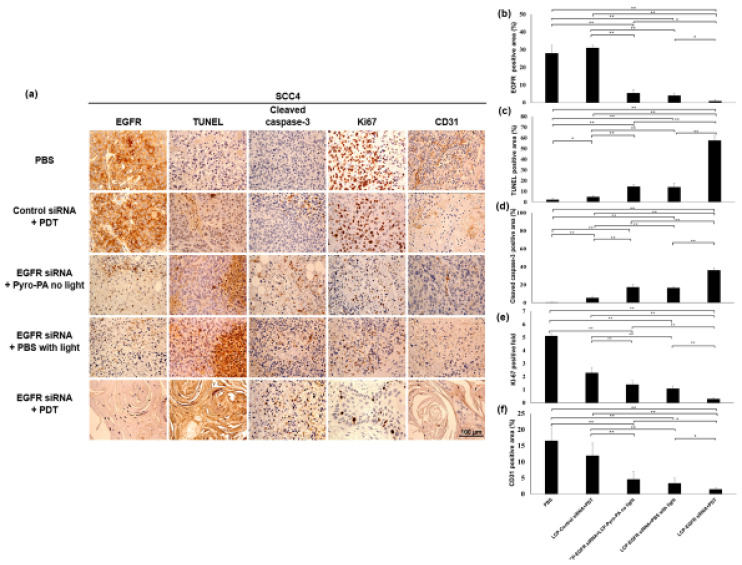

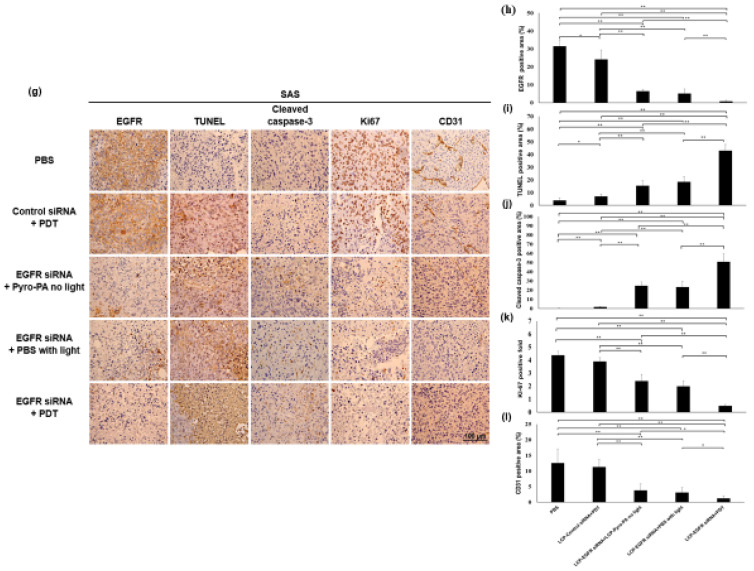
Immunohistochemistry of SCC4 and SAS Tumors; SCC4 tumor cell proliferation and apoptotic cells. Phosphoylated-EGFR (**a**), TUNEL, cleaved caspase-3, Ki-67 and CD31 assays for SCC4 tumor tissue sections. Original magnification 40×. Scale bar stands for 100 μm. Quantitative analysis for Phosphoylated-EGFR (1092) (**b**), TUNEL (**c**), Cleaved Caspase-3 (**d**), Ki-67 (**e**) and CD31 (**f**). SCC4 tumor cell proliferation and apoptotic cells. Phosphoylated-EGFR (**g**), TUNEL, cleaved caspase-3, Ki-67 and CD31 assays for SAS tumor tissue sections. Original magnification 40×. Scale bar stands for 100 μm. Quantitative analysis for phosphoylated-EGFR (1092) (**h**), TUNEL (**i**), cleaved caspase-3 (**j**), Ki-67 (**k**) and CD31 (**l**). Columns, mean (7 images); * *p* < 0.05; ** *p* < 0.01.

The following assays were conducted on SCC4 or SAS tumors for the EGFR marker: Ki67 marker was used for cell proliferation staining; CD31 marker was used for microvessel formation; cleaved caspase-3 was used to mark tumor cell apoptosis and TUNEL was used to stain DNA fragmentation. All of these assays were evaluated and quantified using immunohistochemistry staining (IHC) (Figure 7b–f,h–l). EGFR expression was observed at high levels with tumors of the PBS and LCP siControl+PDT groups, suggesting it was highly expressed in SCC4 and SAS cells. Both SCC4 and SAS tumors showed higher EGFR protein expression levels in the PBS and LCP siControl+PDT groups than in the three siEGFR-loaded LCP NPs groups. These three groups treated with LCP siEGFR nanoparticles showed lower EGFR protein expression (Figure 7b,h), which suggests that EGFR genes were silenced. This EGFR gene knockdown effect could lead to tumor cell apoptosis, DNA fragmentation, and reduced microvessel blood vessel formation, especially with the LCP siEGFR NPs+PDT combined therapy. For the LCP Pyro PA NPs–mediated PDT, LCP siEGFR, and both combined treatments groups, there were increased caspase-3 levels, representing apoptotic cells. Significant differences in the expression of TUNEL positive cells and cleaved caspase-3 between all treatment groups compared to the PBS group (*p* < 0.01, *p* < 0.05) were found in both SCC4 and SAS xenograft models (Figure 7c,d,i,j). The combined LCP siEGFR+PDT group showed significantly drastic cell destruction effects that led to 55% and 40% cell apoptosis detected via TUNEL assay (Figure 7c,i) and 35% and 50% cleaved caspase-3 (Figure 7d,j) expression in SCC4 and SAS models, respectively. Moreover, the LCP siEGFR+LCP-Pyro PA–mediated PDT showed a significant difference from LCP siControl+PDT, indicating that the LCP siEGFR NPs enhanced the apoptotic effect on cancer cells when combined with LCP Pyro PA–mediated PDT in SCC4 and SAS xenograft models. The quantified positive field of Ki67 cells indicated a stronger inhibition effect in the LCP siEGFR+PDT group compared to PBS, LCP siControl+PDT, LCP siEGFR+LCP Pyro PA without light, and LCP siEGFR+PBS+light groups (*p* < 0.01) for SCC4 (Figure 7e) and SAS (Figure 7k). This showed that the combination of LCP siEGFR NPs and LCP Pyro PA–mediated PDT enhanced the inhibition effect of the cell proliferation activity significantly. LCP siControl+PDT, LCP siEGFR+LCP-Pyro PA without light, and LCP siEGFR+PBS+light groups demonstrated significant inhibition (*p* < 0.01) in Ki67 amounts compared to PBS group for SCC4 and SAS xenograft models, revealing that LCP-Pyro PA–mediated PDT alone or siEGFR-loaded LCP NPs alone contributed significantly decreasing tumor cell proliferation. The quantified CD31 microvessel intensities of SCC4 and SAS tumors (Figure 7f,l) showed a dramatic decrease in groups treated with LCP siEGFR NPs for SCC4 and SAS models compared with PBS group (*p* < 0.01 for both). These groups exhibited significant differences compared with the LCP siControl+PDT (*p* < 0.01) group, suggesting that LCP siEGFR NPs showed a positive impact on the inhibition of microvasculature formation of the HNSCC tumors.

### 3.6. Toxicity and Inflammatory Cytokine Studies In Vivo

**Figure 8:**
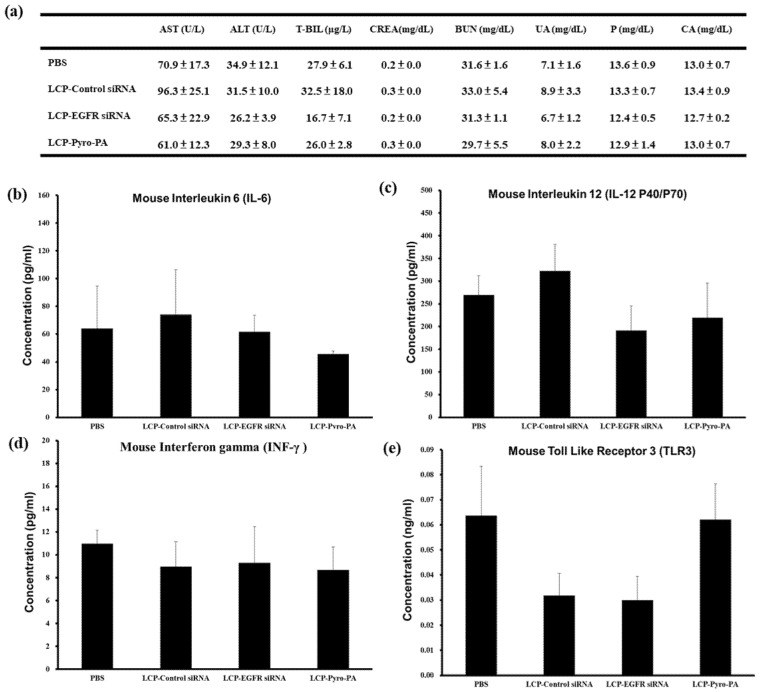
In vivo toxicity and inflammatory response. (**a**) The serum levels of C57BL/6 mice for liver Figure 6. Mouse Interleukin 6 (IL-6) (**b**), interleukin-12 p40/70 (**c**), interferon gamma (**d**), Toll-like receptor 3 (**e**). Data presented as mean ± SD (*n* = 3), * *p* > 0.05 compared to PBS (control) group.

The siEGFR-loaded LCP NPs, siControl-loaded LCP NPs and LCP-Pyro PA NPs were investigated for liver and kidney toxicity. C57BL/6 mice were used for the studies. Twelve mice were distributed into four groups that were given three IV infusions of PBS, LCP Control siRNA NPs, LCP EGFR siRNA NPs and LCP-Pyro PA NPs for three days, one injection per day. Mice were sacrificed by cardiac puncture to collect the blood for assays on the fourth day. For liver function, there was no significant difference in aspartate aminotransferase (AST), alanine aminotransferase (ALT), and total-bilirubin (T-BIL) (*p* > 0.05) for LCP siControl, LCP siEGFR, and LCP Pyro PA groups compared to the PBS group. This suggests no liver function damage. Similarly in the kidney index, the creatinine (CREA), blood urea nitrogen (BUN), uric acid (UA), phosphorus (P), and calcium (CA) amounts indicated no significant effect (*p* > 0.05) (Figure 8a). No abnormal effect of LCP NPs on kidney function was noted, even though their cores are made of calcium phosphate. In Figure 8b–e, no significant difference was observed on the levels of interleukin 6 (IL-6), interleukin 12 (IL-12 P40/P70), interferon gamma (INF-γ), and Toll-like receptor 3 (TLR3) in triplicate (*p* > 0.05) for all groups (PBS, LCP siControl, LCP siEGFR, and LCP Pyro Pa). This further indicates no toxicity resulting from significant activation by LCP NPs of IL-6, IL-12, INF-γand TLR3 in mouse serum (*p* > 0.05). These in vivo toxicity assays suggest that both novel LCP Pyro PA NPs and LCP siRNA NPs are safe based on SCC4 and SAS animal model studies (Figure 8b–e).

## 4. Discussion

Combined therapy has become the mainstream present-day strategy for treating head and neck cancer patients. Clinical practices for head and neck cancer therapy include surgery, chemotherapy and radiotherapy. PDT is a noninvasive modality for cancer therapy with minimum side effects [3,4]. In this study, we were the first to design and formulate novel liposomal Pyro PA nanoparticles with targeted ligand AEAA for photodynamic therapy. The combined treatment of targeted LCP Pyro PA with targeted LCP siRNA NPs in vitro and in vivo showed positive preclinical outcomes. Significantly, this is the first design and application of the novel EGFR siRNA sequence in LCP EGFR siRNA targeting EGFR to human HNSCC. The design mechanism of drug release of the LCP NPs was based on cores of calcium phosphate LCP nanoparticles. LCP NPs are pH-sensitive nanoparticles that release drugs locally and transiently. LCP NPs are quickly taken up into endosomes and release encapsulated Pyro PA or encapsulated EGFR siRNA into cell cytoplasmic compartments [11,14]. The asymmetric bilayer structure of LCP NPs was made of anionic lipids (DOPA), cationic lipids (DOTAP) and cholesterol, and LCP NPs were PEGylated to avoid uptake by the immune system and to extend the circulation period in order to enhance the endocytosis of LCP NPs to cancer cells [11,14]. The second leaflet of LCP NPs was PEGylated and also modified with AEAA target ligands, which target the over-expressed sigma receptors in several kinds of cancer cells, including HNSCC cells [9]. LCP Pyro PA NPs and EGFR siRNA NPs were dispersed evenly with similar particle sizes on TEM photomicrographs (Figure 2 and Figure 3). The particle sizes of targeted EGFR siRNA NPs and targeted LCP Pyro PA NPs averaged 34.9 ± 3.0 nm and 15–20 nm, with zeta potentials of 50.1 ± 1.8 mV and 52.0 ± 7.6 mV, respectively. Cabral et al. compared a range of nanoparticle sizes including 30, 50, 70 and 100 nm for accumulation and effectiveness in several poorly and highly penetrable tumor models. Results suggest that 30~50 nm nanomedicines could be the optimal nanoparticle size to penetrate poorly permeable tumors for achieving a better therapeutic effect [16]. Perrault et al. reported that nanoparticle sizes larger than 100 nm exhibited limited permeation into tumors but could be feasible for antiangiogenic therapy; however, the therapeutic effect on malignant tumors was limited due to the larger nanoparticle sizes [17]. The nanoparticle sizes obtained for both LCP Pyro PA NPs and LCP siEGFR NPs were within the suggested 30~50 nm range to enter into tumor cells via endocytosis [16,17]. From our toxicity studies, the CaP cores were shown to be biodegradable and the double lipid bilayer structure of the nanoparticles was relatively functional with regard to safety, with no observed liver and kidney damage caused by LPC NPs (Figure 8). Edmonds et al. reported that EGFR signaling pathway inhibition leads to enhanced PDT cell toxicity with cell apoptosis upregulation. This suggests that targeting EGFR pathways could be a promising solution for PDT clinical trials for patients with cancer metastasis [18]. Protocols of treatment sequence were investigated in vitro to obtain the best feasible order of therapy, which involved conducting LCP siEGFR NPs gene therapy first, followed by PDT (data not shown). We proved that our novel EGFR siRNA sequence did silence EGFR expression. Combined treatment was implemented to apply siEGFR by IV infusion of LCP siRNA NPs for one injection/day for three days, then conducting LCP Pyro PA NPs–mediated PDT on the fourth day. The therapeutic outcome of PDT was demonstrated in the siControl+PDT group. LCP Pyro PA–mediated PDT enhanced inhibition of tumor proliferation and increased tumor apoptosis. In the incomplete PDT treatment groups (LCP siEGFR+LCP Pyro PA without light and LCP siEGFR+PBS+light), the anti-tumorigenic effects of the LCP siEGFR NPs were observed as LCP siEGFR was effectively delivered to cancer cells resulting in lower levels of EGFR mRNA and protein expression. Significant reduction in tumor volume and cell proliferation (Ki67) and upregulation in cell apoptosis (TUNEL and cleaved caspase-3) were observed. LCP siEGFR gene therapy functioned effectively in vivo. Overall, the combination treatment of siEGFR-loaded LCP NPs and LCP Pyro PA mediated PDT demonstrated the greatest inhibition of tumor volume while maintaining inhibition effect. For combined therapy, mRNA and protein expression of EGFR were significantly inhibited, and consistent results in EGFR immunohistochemical stains were documented. The inhibition of EGFR protein expression was consistent with a decrease in micro-vessel density (CD31 biomarker), which may have been caused by the decrease in Ki67 protein tumor proliferation and enhanced tumor apoptosis (cleaved caspase-3 protein and TUNEL data). In a previous study, liposomal porphysomes were previously formed using pyropheophorbide, with a nanoparticle size of 100nm. Porphysomes have large, tunable extinction coefficients and are effective agents for either photothermal or photoacoustic application [17,18]. In the past, porphysomes were intended for use as a photosensitizer for tumors irradiated under PDT conditions. However, porphysomes did not induce PDT–mediated effects on tissue damage, indicating that porphysomes were ineffective for PDT [18]. Cho et al. investigated a nanocomplex containing dextran sulfate and poly-L-arginine-based polyelectrolyte to deliver EGFR siRNA in a HNSCC xenografted mouse model. The size of this polyelectrolyte nanocomplex was less than 200 nm with a positive zeta potential surface charge. Results showed an enhanced EGFR siRNA uptake efficiency in EGFR gene silencing–tumor cells as well as tumor growth inhibition [19]. However, Cabral et al. reported that nanoparticle size is crucial to the therapeutic success of drug delivery to tumor cells [16]. Nanoparticle sizes less than 50 nm can pass through poorly permeable tumors. These findings suggest that LCP NPs are a better nanodelivery system at <50 nm sizes, promoting effective drug delivery and payloads released to targeted cancer cells. In this study, LCP Pyro PA NPs and LCP siEGFR NPs were both within 20–50 nm in size. A PDT effect was observed for the in vivo LCP-control siRNA+PDT group compared to the PBS group (*p* < 0.01). Most importantly, LCP Pyro PA NPs enhanced EGFR siRNA gene therapy, resulting in a significant decrease in tumor volume (*p* < 0.01) compared with the four other experiment groups (Figure 5).

## 5. Conclusions

In summary, self-designed siEGFR loaded into LCP NPs and combined with novel LCP Pyro PA NPs–mediated PDT promises to improve the treatment effectiveness in HNSCC. This novel nanomedicine combined targeted LCP Pyro PA mediated PDT and gene therapy in order to effectively silence the EGFR oncogene marker.

## Data Availability

Not applicable.
